# The Influence of Calcium Ions and pH on Fluoride Release from Commercial Fluoride Gels in an In Vitro Study

**DOI:** 10.3390/gels11070486

**Published:** 2025-06-23

**Authors:** Paweł J. Piszko, Michał Kulus, Aleksandra Piszko, Jan Kiryk, Sylwia Kiryk, Julia Kensy, Agata Małyszek, Mateusz Michalak, Wojciech Dobrzyński, Jacek Matys, Maciej Dobrzyński

**Affiliations:** 1 Department of Biomedical Engineering, Faculty of Fundamental Problems of Technology, Wrocław University of Science and Technology, Wybrzeże Wyspiańskiego 27, 50-370 Wrocław, Poland; 2 Division of Ultrastructural Research, Wroclaw Medical University, Chałubińskiego 6a, 50-368 Wrocław, Poland; 3 Department of Pediatric Dentistry and Preclinical Dentistry, Wroclaw Medical University, Krakowska 26, 50-425 Wroclaw, Polandmaciej.dobrzynski@umw.edu.pl (M.D.); 4 Dental Surgery Department, Wroclaw Medical University, Krakowska 26, 50-425 Wroclaw, Poland; 5 Faculty of Dentistry, Wroclaw Medical University, Krakowska 26, 50-425 Wroclaw, Poland; 6 Department of Biostructure and Animal Physiology, Wrocław University of Environmental and Life Sciences, Kożuchowska 1, 51-631 Wroclaw, Poland; 7 Medical Center of Innovation, Wroclaw Medical University, Krakowska 26, 50-425 Wroclaw, Poland; 8 Department of Dentofacial Orthopedics and Orthodontics, Division of Facial Abnormalities, Wroclaw Medical University, Krakowska 26, 50-425 Wroclaw, Poland

**Keywords:** fluoride release, fluoride gels, calcium interference, in vitro incubation, dental biomaterials, statistical analysis

## Abstract

Fluoride gels are widely used in dental prophylaxis due to their proven ability to prevent demineralization and promote remineralization of hard dental tissues. However, the effectiveness of fluoride release from such gels may be significantly influenced by environmental factors such as pH and the presence of calcium ions. This in vitro study aimed to evaluate how these variables affect fluoride ion release from three commercially available fluoride gels—Clarben, Flairesse, and Lunos. The gels were incubated in artificial saliva of varying pH levels (4.5, 6.0, 7.0, and 7.5) with and without the addition of calcium, as well as in other water-based media—tap water, deionized water, and 0.9% NaCl solution. Fluoride release and changes in pH were measured and statistically analyzed using a multifactorial ANOVA. The results revealed that fluoride release was highest in calcium-free environments and at neutral to slightly alkaline pH, while the presence of calcium significantly reduced fluoride availability. Among the tested products, Flairesse and Lunos exhibited sensitivity to calcium’s presence, unlike Clarben. Fluoride release was generally higher in water than in artificial saliva. Additionally, all gels induced a decrease in pH, which varied depending on the initial pH and calcium content. These findings underline the importance of environmental conditions in optimizing the clinical efficacy of fluoride gel applications.

## 1. Introduction

**Figure 1:**
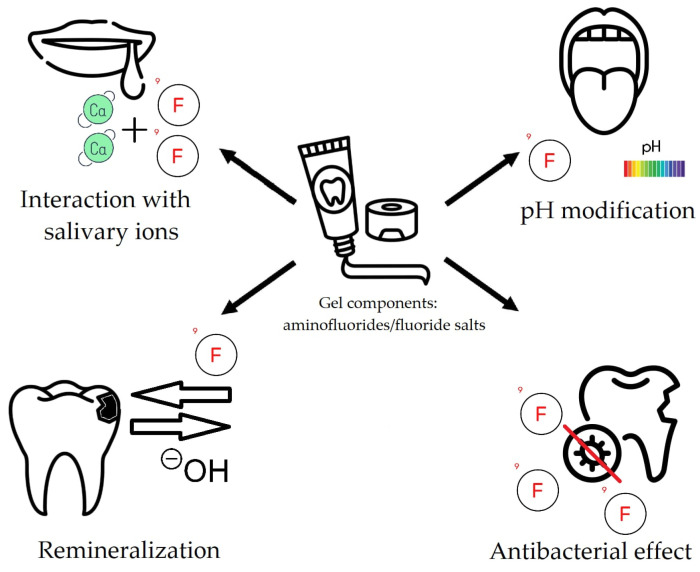
Multimodal operation of fluoride gels in dentistry (created with Freepik.com).

The use of fluoride in dental prophylaxis has been shown to be highly beneficial. The metabolism of plaque bacteria is influenced by fluoride, which has been shown to inhibit enolase activity. This, in turn, reduces the amount of acid produced. It also affects the transport of glucose by bacteria, which blocks the intracellular storage of polysaccharides. The above-mentioned effects result in the inhibition of the growth of cariogenic microorganisms [1,2,3,4]. It is therefore imperative to ensure that the fluoride level in the oral cavity is adequately maintained in order to prevent the occurrence of dental caries. In a water-based environment, fluoride ions diffuse into the inorganic part of the tooth tissues where they react with hydroxyapatite crystals and replace hydroxyl groups, finally leading to the formation of fluorapatite [5], which is more caries-resistant [6,7,8,9]. In comparison with hydroxyapatite, crystals of fluorapatite are more stable in structure, have lower solubility, have superior thermal stability, and are more resistant to acids [3]. Their movement is diffusion-operated (diffusion of fluids into and out of the material and diffusion of fluoride ions into the dental tissues) [10]. In summary, fluoride is indicated as a vital element in maintaining a balance between the demineralization and remineralization of enamel [11]. Furthermore, it has been demonstrated that fluoride exerts antimicrobial properties, chiefly by means of disrupting the metabolism of cariogenic bacteria such as *S. mutans*. It has been demonstrated to inhibit key bacterial virulence factors, including acid production and glucan synthesis. Nevertheless, the clinical relevance of these antimicrobial effects in vivo remains to be fully elucidated [12]. The multimodal action of fluoride gels in dentistry elaborated above is depicted graphically in Figure 1.

Fluoride gels are among the professionally administered topical agents [13]. A systematic review by Twetman et al., including clinical trials, confirmed their efficacy in preventing caries [14]. Dental fluoride gels are commercially available in concentrations ranging from 5000 to 12,300 ppm. They are recommended for use in children over six years of age, and the frequency of treatments is determined based on the patient’s level of caries risk [15]. Low-pH formulations are available, such as acidified phosphate fluoride (APF) gel, along with neutral-pH formulations, such as sodium fluoride gel. APF gel is most commonly used professionally by dentists. It is recommended that patients with porcelain fillings use neutral gels. The rationale behind this recommendation is to avoid the possibility of etching of these fillings by low-pH gels [13].

Researchers are continuously striving to enhance the availability and efficacy of fluoride released from various dental materials. This is driven by the important role fluoride plays in preventing dental caries and supporting enamel remineralization. Numerous scientific studies have focused on evaluating the influence of different factors that may affect the secretion or release of fluoride from these materials. Among the variables considered, the environment into which fluoride is released has emerged as one of the most significant. This environment includes not only the elemental and chemical composition of the surrounding medium but also factors such as the pH, ionic strength, and specific type of medium in which the tested dental material samples are stored during experimentation. The most frequently utilized storage media in fluoride-release studies are demineralized water, deionized water, and artificial saliva. Among these, deionized water is generally regarded as the standard reference medium for assessing the potential of fluoride release under non-stimulated, controlled laboratory conditions [16,17,18,19,20,21]. Artificial saliva is used to simulate the natural oral environment, although exact reproduction of human saliva’s properties is impossible due to the inconsistent and unstable nature of natural saliva. Therefore, the development of artificial saliva is essential for well-justified and controlled experiments [22]. It is also hypothesized that the presence of calcium ions (Ca^2+^) in the environment may affect fluoride release by forming insoluble compounds that limit fluoride availability [23,24,25]. This assertion is corroborated by the observation that the placement of material samples in solutions containing calcium concomitantly results in a decline in their ion content [26,27]. For instance, when fluoride ions are released from dental materials into the oral environment or a testing medium, they have the capacity to react with calcium ions to form calcium fluoride (CaF_2_). This reaction occurs when fluoride ions are released from dental materials into the oral environment or testing medium. However, the solubility of calcium fluoride increases as the pH decreases. Consequently, fluoride ions exhibit increased availability at lower pH levels in the presence of calcium cations. At elevated pH levels, the formation of the insoluble compound calcium fluoride becomes more dominant, trapping fluoride and reducing its bioavailability.

The main objective of this study was to determine the effect of calcium ions and pH on fluoride release from commercial fluoride gels for teeth during an in vitro study. It is important to note that numerous studies have been conducted on dental filling materials such as glass ionomers, compomers, or composites, but this topic has not been addressed for fluoride gels. Moreover, the novelty of the conducted study is the evaluation of fluoride release from three commercial gels in four media (tap water, demineralized water, 0.9% NaCl solution, and artificial saliva with and without calcium ions) with different chemical compositions, which had an impact on the study results. The frequent use of gels in caries prevention generates the need to determine the above-mentioned effect and makes the results of this study potentially clinically relevant.

## 2. Results and Discussion

### Results of Statistical Analysis of Fluoride Release and pH Assessment

**Figure 2:**
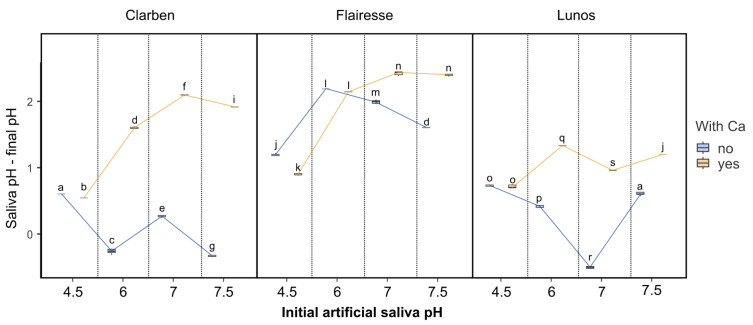
The box plots depict the difference between the initial saliva pH and the pH measured after the gel was applied. Boxes marked with different letters are statistically different (*p* < 0.01 in the Tukey post hoc test), while those with at least one letter in common are not significantly different. The post hoc test included pairwise comparisons of all possible combinations of initial pH, gels, and the presence of Ca^2+^.

Figure 2 shows box plots depicting changes in saliva pH in relation to the initial pH of artificial saliva and the presence (or absence) of Ca for the three evaluated gels. For the lowest artificial saliva pH (4.5), the change in pH is only slightly dependent on the presence of Ca, regardless of the applied gel. For a higher pH of artificial saliva, the decrease in pH is more pronounced in saliva devoid of Ca. The exception to this is Flairesse gel, for which the pH change is less affected by the presence of Ca than it is for the other two gels. According to the multifactorial ANOVA (Table 1), all categorizing variables and their interactions have high ƞ^2^ values (>0.9). This reflects the irregular patterns of pH change among the different gels and artificial saliva compositions. Such pronounced interactions suggest complex underlying mechanisms governing the behavior of the gels in different saliva environments.

**Figure 3:**
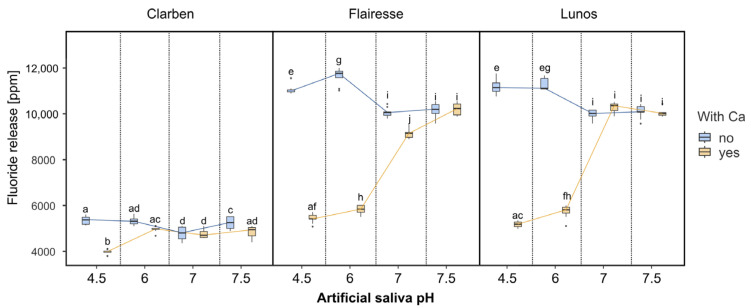
The box plots depict the concentration of fluoride released after gel application. Boxes marked with different letters are statistically different (*p* < 0.01 in the Tukey post hoc test), while those with at least one letter in common are not significantly different. The post hoc test included pairwise comparisons of all possible combinations of initial pH, gels, and the presence of Ca^2+^.

Figure 3 presents box plots depicting fluoride release depending on the gel and the pH and calcium presence in artificial saliva. Although Clarben has the lowest fluoride concentration, its fluoride release is relatively stable and almost independent of pH or calcium presence. Except for an outlier result for saliva with a pH of 4.5 and calcium, the mean fluoride release from Clarben for all pH and Ca^2+^ presence combinations ranges from 4764 to 5344 ppm. Flairesse and Lunos exhibit a different pattern: at pH 4.5 and 6, the fluoride release is approximately half as much in saliva with Ca, and at pH 7.0–7.5, the fluoride release is similar in saliva with or without Ca. The multifactorial ANOVA ƞ^2^ value for main effects is highest for the gel type (see Table 1). Regarding interactions, the presence of calcium combined with saliva pH is indicated as the most influential interaction factor in differential fluoride release.

To compare fluoride release in water and saliva, three types of aqueous environments were used: tap water, demineralized water, and demineralized water with the addition of 0.9% sodium chloride (NaCl). After evaluating the results, it was determined that there were no notable or statistically significant differences in fluoride release between demineralized water alone and demineralized water containing NaCl. Because of this lack of distinction, both types were grouped together and treated as a single category for the purpose of analysis, simplifying the comparison. In order to minimize the potential impact of varying pH levels on fluoride release, only saliva samples with neutral to slightly basic pH values—specifically pH 7.0 and 7.5—were included in the comparative analysis. In the context of this study, tap water was defined as water that contains calcium ions, while demineralized water was considered to be water that lacks calcium.

**Figure 4:**
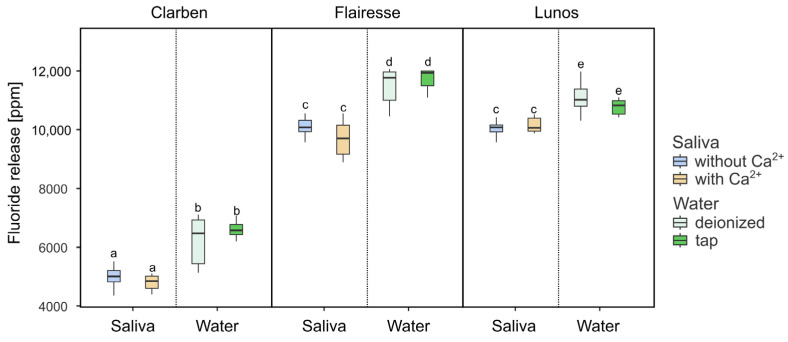
Box plots depicting fluoride release in artificial saliva and water. Boxes marked with different letters are statistically different (*p* < 0.01 in the Tukey post hoc test), while those with at least one letter in common are not significantly different. The post hoc test included pairwise comparisons of all possible combinations of initial pH, gels, and the presence of Ca^2+^.

The outcomes of the study are presented in Figure 4. A high effect size (ƞ^2^) was observed only in relation to the different gels tested, as shown in Table 1. No other main effects or interaction effects significantly influenced the level of fluoride released. In general, the evaluated gels released slightly less fluoride in saliva compared to water, regardless of the presence or absence of calcium ions.

Studies show that fluoride release values in deionized water and artificial saliva differ significantly [18,28,29,30,31]. It was observed that higher values of fluoride were present in deionized water, which was also confirmed in the presented evaluation (Figure 4). A decline in values was observed in the artificial saliva samples. This phenomenon may be attributable to the presence of cations and anions within the artificial saliva sample, which may exert an ionic effect on the solubility of the material under investigation [23]. The interactions between ions in fluoride gels such as Clarben, Flairesse, and Lunos may involve not only the active fluoride carrier (NaF) but also the other components present in their formulations (see Table 3). These additional ingredients could potentially influence the behavior and release profile of fluoride ions. However, the specific mechanisms underlying these interactions are beyond the scope of this study.

Another critical factor affecting ion release is the composition of the gel and the structural properties of its matrix, which determine fluoride ion containment and diffusion. While all of the gels that were evaluated shared NaF as the active ingredient, with a consistent fluoride ion concentration of 12,300 ppm, their distinct formulations, as outlined in Table 3, suggest that there are variations in the components of the excipients that may affect the kinetics of dissolution and the availability of the ions in a differential manner. However, this study did not investigate how these compositional differences in matrix structure might specifically modulate fluoride release dynamics.

In commonly available gels, fluoride occurs in an organic form, e.g., in amine fluorides, and/or is a component of salts such as NaF, KF, and SnF_2_ [32,33]. Studies show that gels containing amine fluorides are a more efficient source of fluoride. This is due to differences in reactivity between cations and anions contained in solvents and fluoride carriers. The fluoride ions released from the dissolved salt react very quickly with other elements present in water or saliva, such as calcium, precipitating as CaF_2_. In turn, amine fluorides show greater stability and a lack of interactions with other ions, which makes this form more effective in preventing caries [34,35]. The influence of the type of matrix on changes in salivary pH parameters is also significant. It was noted that Fluormex gel containing amine fluoride and sodium fluoride led to a greater decrease in the pH value than Fluor Protector gel with potassium fluoride. Moreover, the greatest decrease, to pH = 4.07 after 48 h of incubation, among all physiological solutions used was noted in artificial saliva, a solvent that creates conditions most similar to those prevailing in the oral cavity. In the case of Fluor Protector gel, the pH value decreased to 5.54 [36].

The findings of these studies indicate that in a lower-pH environment, there is an increased degree of fluoride gel structure relaxation, which, in turn, facilitates the release of fluoride ions. A study by Wiglusz et al., conducted on nanohydroxyapatite hydrogels, demonstrated that fluoride release is significantly greater at a pH of 4.5 than at pH 6.6 or 8.0 [37]. Conversely, the present study documented optimal fluoride release in pH conditions that are less conducive to demineralization. Furthermore, in environments that are neutral or slightly alkaline, the presence of calcium has been shown to impede a rapid decline in pH following the application of fluoride gel. This is of significance because, while calcium generally reduces fluoride availability, it may indirectly contribute to stabilizing the pH environment within a safer range. In conclusion, the present study demonstrated that although gels reduce the pH level, their optimal efficacy in fluoride release transpires under conditions that exceed the threshold for enamel demineralization, signifying a multifaceted relationship between these factors.

Shakeel et al., in an in vivo study on rats, noted that the pH of the gel itself is also of vital importance. The use of gels acidified with 1.23% acidulated phosphate fluoride (APF) leads to the greatest decrease in pH = 4.0 when compared to gels with 0.2% NaF and 0.4% SnF_2_ [38]. The critical pH below which hydroxyapatite dissolves is 5.5, whereas for fluorapatite it is below 4.5. While an acidic environment facilitates the incorporation of fluoride into enamel’s mineral structure by increasing its surface area, it is important to recognize that low pH values in saliva can lead to enamel demineralization [36,39].

In all three gels tested, fluoride is present in the form of a 12,300 ppm NaF matrix; despite this, differences in the amount of its release were observed. Under the same pH and solution conditions, Flairesse and Lunos gels released even twice as much fluoride as Clarben, which, in contrast to them, regardless of the presence of calcium ions and pH changes, showed the highest release stability. The above may indicate that other ingredients contained in the gels may also have an impact on the amount and dynamics of fluoride release.

Furthermore, it is noteworthy that Clarben contains the highest number of ingredients in its composition when compared to Flairesse and Lunos (Table 3). This formulation was associated with lower, but more stable, fluoride release (Figure 4). However, a detailed analysis of the impact of individual components was not performed; therefore, this observation is supported not by specific analytical data but by the amount of released fluoride.

## 3. Conclusions

The results of this study lead to the conclusion that calcium, the pH of artificial saliva, and the type of incubation fluid, as well as the type of gel itself, affect the release of fluoride from fluoride gels. Provided that calcium ions are absent, the gel’s efficiency may be enhanced when an appropriate acidic environment is maintained, as such ions have the capacity to diminish the amount of fluoride released by as much as twofold. In a neutral or slightly alkaline environment, calcium can prevent an undesirable rapid drop in pH after the use of fluoride gel. It is worth noting that more fluoride was released into water than into artificial saliva, which may prove that the presence of other ions can also alter the amount of fluoride released from the gel. The above confirms the significant influence of many factors on the amount and dynamics of fluoride release from gels.

## 4. Materials and Methods

### 4.1. Liquids Used for Incubation

The release of fluoride and the assessment of pH were conducted in eleven distinct liquids. Specifically, the following substances were used: tap water, demineralized water, a 0.9% NaCl solution, and artificial saliva (pH = 4.5, 6.0, 7.0, and 7.5) with and without the addition of calcium ions. The solutions of NaCl and artificial saliva were based on demineralized water. To simulate oral conditions, two types of artificial saliva were utilized: one containing calcium ions and one without. Both formulations were based on established protocols commonly used in in vitro studies of dental materials [40,41]. The artificial saliva was prepared using demineralized water and a mixture of electrolytes and organic components, including urea, sodium chloride (NaCl), potassium chloride (KCl), sodium dihydrogen phosphate dihydrate (NaH_2_PO_4_·2H_2_O), and sodium sulfide nonahydrate (Na_2_S·9H_2_O). The calcium-containing saliva also included calcium chloride dihydrate (CaCl_2_·2H_2_O). The origin, detailed composition, and calcium concentration of the liquids used are presented in Table 2.

### 4.2. Evaluated Fluoride Gels

**Figure 5:**
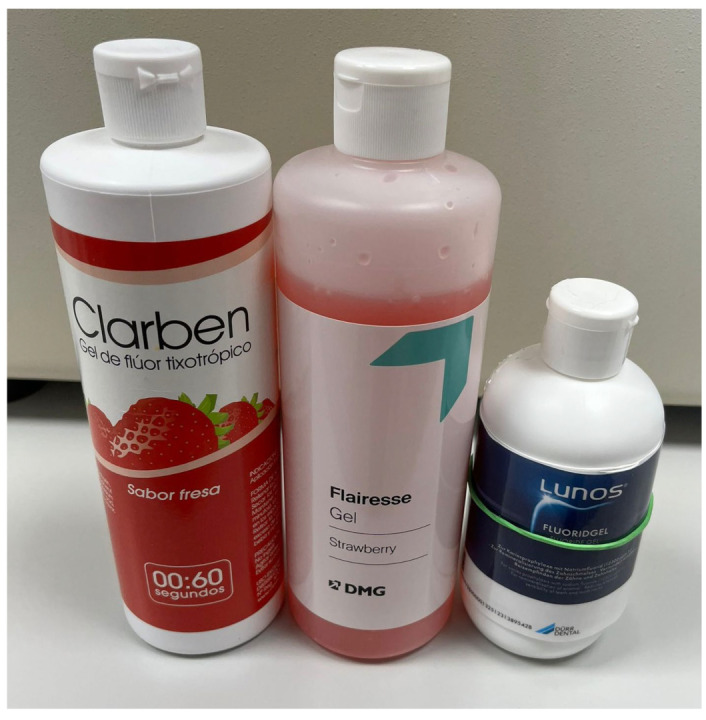
Clarben, Flairesse, and Lunos—commercial fluoride gels evaluated in the study.

Studies were conducted on three commercially available fluoride gels: Thixotropic Fluoride Gel—Strawberry Flavour (Laboratorios Clarben, Madrid, Spain), Flairesse Gel Strawberry (DMG Chemisch-Pharmazeutische Fabrik GmbH, Hamburg, Germany), and Lunos Fluoride Gel (DÜRR DENTAL SE, Bietigheim-Bissingen, Germany). The three fluoride gels—Clarben, Flairesse, and Lunos—were selected based on their comparable fluoride concentration (12,300 ppm NaF), commercial availability, and popularity in clinical use across Europe. This selection enabled us to study how environmental factors such as the pH and calcium ion concentration influence fluoride release while minimizing confounding differences related to fluoride content. The fluoride gels utilized in the presented study are depicted in Figure 5, and their compositions and concentrations of fluoride are presented in Table 3.

**Table 3:** Manufacturers, lot/batch numbers, compositions, and fluoride concentrations of evaluated fluoride gels.

Name of the Product (Abrr.)	Manufacturer	Lot/Batch Number	Composition	Fluoride Form and Amount	Ref.
Clarben	Clarben, Madrit, Spain	D240325	Purified water, FD & Red no. 40, saccharin sodium, sodium benzoate, titanium dioxide, alpha-tocopherol acetate, citric acid monohydrate, xylitol, magnesium aluminum silicate, xanthan gum, phosphoric acid, hydrofluoric acid, polysorbate 20	NaF 12,300 ppm	[42]
Flairesse	DMG Dental, Hamburg, Germany	304852	Water, carboxymethyl cellulose, sodium fluoride, phosphoric acid, xylitol, additives	NaF 12,300 ppm	[43]
Lunos	Dürr Dental, Bietigheim-Bissingen, Germany	389542	Water, sorbitol, sodium fluoride, disodium hydrogen phosphate, hydrogenated castor oil (PEG-40), hydroxyethyl cellulose, phosphoric acid, sodium saccharin, flavor	NaF 12,300 ppm	[44]

### 4.3. Methodology of Fluoride Release and pH Assessment

The fluoride release and pH assessment was conducted in accordance with previously reported methodologies [35,36].

**Figure 6:**
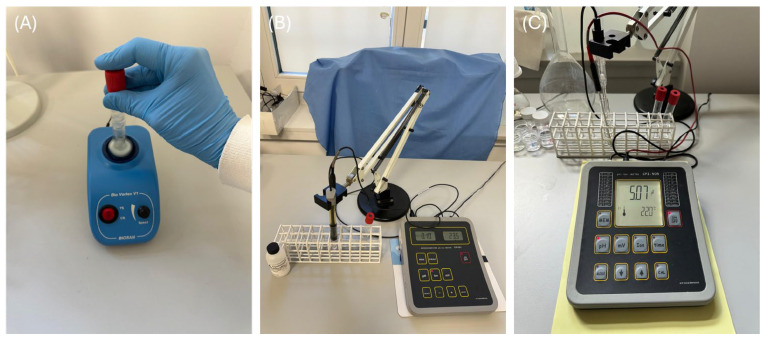
Bio Vortex V1 (**A**), 9609 Orion selective electrode and CPI-551 processing unit (**B**), and ESAgP-303W electrode connected to CPI-505 pH meter (**C**).

An amount of 0.055 g of each of the three evaluated commercial fluoride gels was placed into five separate polypropylene tubes per gel. Subsequently, 5.5 mL of each solution listed in Table 2 was added to the tubes and mixed using a Bio Vortex V1 (Figure 6A, Biosan, Riga, Latvia), then magnetically stirred at a pace of 500 RPM. The concentration of released F^−^ ions was measured using a 9609 Orion selective electrode (Thermo Scientific, Waltham, MA, USA) connected to a CPI-551 processing unit (Figure 6B, Elmetron, Zabrze, Poland). This measurement was taken immediately after the homogenization process. The mean values of fluoride obtained in the current study are presented in Table S1. The pH of the incubation solutions was measured both before incubation and after the fluoride release assessment using an ESAgP-303W electrode (Eurosensor, Gliwice, Poland) connected to a CPI-505 pH meter (Figure 6C, Elmetron). This assessment was conducted in five replicates for each solution (N = 5). The fluoride release was measured, with each measurement being repeated ten times (N = 10).

### 4.4. Statistical Analysis

The analysis performed in the current study used a multifactorial ANOVA to determine the effects of various factors and their interactions on fluoride release and pH changes. The Tukey post hoc test was used to determine the significance of differences between distinct pairs of dependent variables. The statistical analysis was performed using Statistica ver. 13 (TIBCO Software Inc., Palo Alto, CA, USA) and Jamovi version 2.6 [45], which uses the R statistical environment [46]. The statistical significance threshold was established at *p* < 0.01.

## Figures and Tables

**Table 1:** A summary of multifactorial ANOVAs. ƞ^2^ (eta squared) is a measure of effect size utilized in analyses of variance to quantify the proportion of the total variance in the dependent variable that results from a particular factor or their interaction. It signifies the extent to which a factor is associated with the observed outcome. Statistically insignificant values are grayed out.

Effect	*p*-Value	ƞ^2^
Fluoride release		
Intercept	<0.001	0.999
Gel	<0.001	0.988
Ca presence	<0.001	0.960
Initial pH	<0.001	0.864
Gel*Ca presence	<0.001	0.871
Gel*Initial pH	<0.001	0.756
Ca presence*Initial pH	<0.001	0.953
Gel*Ca presence*Initial pH	<0.001	0.882
pH change		
Intercept	<0.001	1.000
Gel	<0.001	0.999
Ca presence	<0.001	0.998
Initial pH	<0.001	0.993
Gel*Ca presence	<0.001	0.996
Gel*Initial pH	<0.001	0.997
Ca presence*Initial pH	<0.001	0.996
Gel*Ca presence*Initial pH	<0.001	0.991
Fluoride release in water vs. in saliva		
Intercept	<0.001	0.997
Water/Saliva	<0.001	0.663
Gel	<0.001	0.959
Ca presence	0.6	0.001
Water/Saliva*Gel	<0.001	0.138
Water/Saliva*Ca presence	0.022	0.023
Gel*Ca presence	0.41	0.008
Water/Saliva*Gel*Ca presence	<0.001	0.062

**Table 2:** Juxtaposition of incubation solutions used during experiments, including their pH, calcium concentration, and composition/origin.

No.	Solution	pH [a.u.]	Concentration of Ca^2+^ [mg/mL]	Origin/Composition/Supplier
1	Tap water	8.08	0.0860 *	Sourced from ul. Chałubińskiego 3, Wrocław, Poland
2	Demineralized water	6.51	0.0	Stapar (Żnin, Poland)
3	NaCl *	6.96	0.0	0.9% NaCl solution based on demineralized water from pt.2
4	Artificial saliva with calcium ions **	4,5; 6.0; 7.0; 7.5	0.2476	Solution based on demineralized water from pt.2 containing 1 g/L of urea, 0.4 g/L of NaCl, 0.4 g/L of KCl, 0.908 g/L of CaCl_2_·2H_2_O, 0.78 g/L of NaH_2_PO_4_·2H_2_O, and 0.005 g/L of Na_2_S·9H_2_O
5	Artificial saliva without calcium ions **	4,5; 6.0; 7.0; 7.5	0.0	Solution based on demineralized water from pt.2 containing 1 g/L of urea, 0.4 g/L of NaCl, 0.4 g/L of KCl, 0.78 g/L of NaH_2_PO_4_·2H_2_O, and 0.005 g/L of Na_2_S·9H_2_O

* The concentration of calcium ions (Ca^2+^) in tap water was determined using data provided by Wrocław Municipality Waterworks (https://www.mpwik.wroc.pl/strefa-klienta/uslugi/uslugi-laboratoryjne/parametry-wody/ accessed on 4 June 2025). ** The following substances were manufactured by Chempur (Piekary Ślaskie, Poland): sodium chloride (NaCl), potassium chloride (KCl), sodium bisulfate (Na_2_S·9H_2_O), sodium hydrogen phosphate (NaH_2_PO_4_·2H_2_O), and urea. Calcium chloride dihydrate (CaCl_2_·2H_2_O) was supplied by Sigma Aldrich (St. Louis, MO, USA).

## Data Availability

Data are contained within the article.

## Supplementary Materials

The following supporting information can be downloaded at https://www.mdpi.com/article/10.3390/gels11070486/s1, Table S1: Mean fluoride release for all parameter combinations used in the current study, ranked from highest to lowest.
