# Supplementary material for: The Influence of Calcium Ions and pH on Fluoride Release from Commercial Fluoride Gels in an In Vitro Study
Source: Gels. 2025 Jun 23;11(7):486. doi: 10.3390/gels11070486 (PMC12296090; doi:10.3390/gels11070486)
Supplement: Supplementary file 1 [file gels-11-00486-s001.zip › gels-3711645-supplementary.pdf]

Influence of Calcium Ions and pH on Fluoride Release from Commercial Fluoride Gels in an In Vitro Study

Supplementary Materials

Paweł J. Piszko [1,*], Michał Kulus [2], Aleksandra Piszko [3], Jan Kiryk [4], Sylwia Kiryk [3], Julia Kensy [5], Agata Małyszek [6], Mateusz Michalak [7], Wojciech Dobrzyński [8], Jacek Matys [4,*] and Maciej Dobrzyński [3]

[1] Department of Biomedical Engineering, Faculty of Fundamental Problems of Technology, Wrocław University of Science and Technology, Wybrzeże Wyspiańskiego 27, 50-370, Poland
[2] Division of Ultrastructural Research, Wroclaw Medical University, Chałubińskiego 6a, 50-368 Wrocław, Poland
[3] Department of Pediatric Dentistry and Preclinical Dentistry, Wroclaw Medical University, Krakowska 26, 50-425 Wroclaw, Poland
[4] Dental Surgery Department, Wroclaw Medical University, Krakowska 26, 50-425 Wroclaw, Poland
[5] Faculty of Dentistry, Wroclaw Medical University, Krakowska 26, 50-425 Wroclaw, Poland
[6] Department of Biostructure and Animal Physiology, Wrocław University of Environmental and Life Sciences, Kożuchowska 1, 51-631 Wroclaw, Poland
7 Medical Center of Innovation, Wroclaw Medical University, Krakowska 26, 50-425 Wroclaw, Poland
[8] Department of Dentofacial Orthopedics and Orthodontics, Division of Facial Abnormalities, Wroclaw Medical University, Krakowska 26, 50-425 Wroclaw, Poland

***Correspondence: pawel.piszko@pwr.edu.pl (P.J.P); maciej.dobrzynski@umw.edu.pl (M.D.)**

Table S1. Mean fluoride release for all parameters combination used in the current study, ranked from highest to lowest.

Group	Mean fluorine [ppm]	SD
Tap water, Flairesse	11703.90	378.121
Artificial saliva without Ca2+ pH = 6.0, Flairesse	11633.70	335.356
Demineralized water , Lunos	11302.80	378.731
Artificial saliva without Ca2+ pH = 6.0, Lunos	11281.10	252.064
Artificial saliva without Ca2+ pH = 4.5, Lunos	11197.10	335.140
Artificial saliva without Ca2+ pH = 4.5, Flairesse	11044.60	191.264
Tap water, Lunos	10759.90	258.658
0.9% NaCl, Lunos	10544.50	216.767
Artificial saliva with Ca2+, pH = 7.0, Lunos	10270.50	229.817
Artificial saliva with Ca2+, pH = 7.5, Flairesse	10196.20	268.635
Artificial saliva without Ca2+ pH = 7.5, Flairesse	10133.60	343.986
Artificial saliva without Ca2+ pH = 7.5, Lunos	10102.20	267.735
Artificial saliva without Ca2+ pH = 7.0, Flairesse	10056.20	188.782
Artificial saliva with Ca2+, pH = 7.5, Lunos	10028.40	156.844
Artificial saliva without Ca2+ pH = 7.0, Lunos	9988.30	196.122
Artificial saliva with Ca2+, pH = 7.0, Flairesse	9124.20	200.358
Demineralized water, Clarben	6808.50	250.833

Tap water, Clarben	6636.30	288.927
Artificial saliva with Ca2+, pH = 6.0, Flairesse	5826.60	190.142
Artificial saliva with Ca2+, pH = 6.0, Lunos	5752.70	277.946
Artificial saliva with Ca2+, pH = 4.5, Flairesse	5436.80	189.103
Artificial saliva without Ca2+ pH = 4.5, Clarben	5344.10	172.749
Artificial saliva without Ca2+ pH = 6.0, Clarben	5329.50	173.168
0.9% NaCl, Clarben	5301.10	178.013
Artificial saliva without Ca2+ pH = 7.5, Clarben	5246.60	281.562
Artificial saliva with Ca2+, pH = 4.5, Lunos	5180.30	132.244
Artificial saliva with Ca2+, pH = 6.0, Clarben	4970.90	120.005
Artificial saliva with Ca2+, pH = 7.5, Clarben	4859.20	240.455
Artificial saliva without Ca2+ pH = 7.0, Clarben	4793.10	293.128
Artificial saliva with Ca2+, pH = 7.0, Clarben	4764.90	199.334
Artificial saliva with Ca2+, pH = 4.5, Clarben	3986.00	86.052
Artificial saliva with Ca2+, pH = 7.5, No gel	7.50	0.000
Artificial saliva without Ca2+ pH = 7.5, No gel	7.50	0.000
Artificial saliva with Ca2+, pH = 7.0, No gel	7.00	0.000
Artificial saliva without Ca2+ pH = 7.0, No gel	7.00	0.000
Artificial saliva with Ca2+, pH = 6.0, No gel	6.00	0.000
Artificial saliva without Ca2+ pH = 6.0, No gel	6.00	0.000
Artificial saliva with Ca2+, pH = 4.5, No gel	4.50	0.000
Artificial saliva without Ca2+ pH = 4.5, No gel	4.50	0.000
Tap water, No gel	0.26	0.015
Demineralized water , No gel	0.00	0.000
0.9% NaCl, No gel	0.00	0.000
